# Machine Learning Full NMR Chemical Shift Tensors of Silicon Oxides with Equivariant Graph Neural Networks

**DOI:** 10.1021/acs.jpca.2c07530

**Published:** 2023-03-02

**Authors:** Maxwell C. Venetos, Mingjian Wen, Kristin A. Persson

**Affiliations:** † Department of Materials Science and Engineering, University of California, Berkeley, California 94720, United States; ‡ Department of Chemical and Biomolecular Engineering, University of Houston, Houston, Texas 77204, United States; § Molecular Foundry, Lawrence Berkeley National Laboratory, Berkeley, California 94720, United States

## Abstract

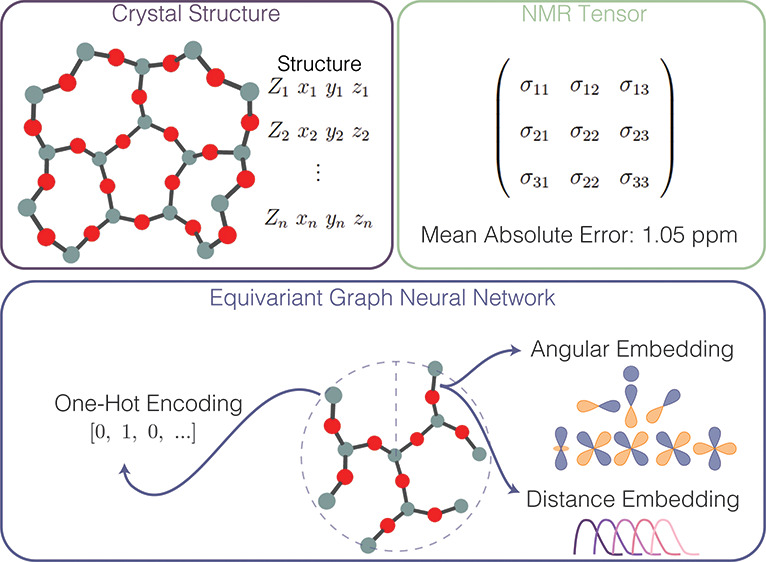

The nuclear magnetic resonance (NMR) chemical shift tensor is a highly sensitive probe of the electronic structure of an atom and furthermore its local structure. Recently, machine learning has been applied to NMR in the prediction of isotropic chemical shifts from a structure. Current machine learning models, however, often ignore the full chemical shift tensor for the easier-to-predict isotropic chemical shift, effectively ignoring a multitude of structural information available in the NMR chemical shift tensor. Here we use an equivariant graph neural network (GNN) to predict full ^29^Si chemical shift tensors in silicate materials. The equivariant GNN model predicts full tensors to a mean absolute error of 1.05 ppm and is able to accurately determine the magnitude, anisotropy, and tensor orientation in a diverse set of silicon oxide local structures. When compared with other models, the equivariant GNN model outperforms the state-of-the-art machine learning models by 53%. The equivariant GNN model also outperforms historic analytical models by 57% for isotropic chemical shift and 91% for anisotropy. The software is available as a simple-to-use open-source repository, allowing similar models to be created and trained with ease.

## Introduction

Many useful properties of materials manifest from the precise structure of a given composition. Traditional structure determination techniques such as X-ray diffraction (XRD) are suitable for atoms of moderate to high atomic number; however, they can lead to ambiguous structure assignments for materials containing atoms with low atomic number.^1^ In addition, XRD relies heavily on long-range order for correct measurement, but such long-range order is often lacking in many classes of materials, e.g., nanostructures, amorphous materials, and materials with a tetrahedral network, making structural characterization of such materials via XRD difficult. Such a class of materials are exemplified by silicates, which consist of a tetrahedral structure of silicons and oxygens (which have low atomic number and are difficult to observe via XRD).

Silicate materials are ubiquitous, from naturally occurring rocks and minerals like quartz^2−5^ and garnet^6^ to diverse manufacturing applications such as glasses,^7,8^ cements,^9^ and zeolite catalysts.^10,11^ To discover new potential applications of silicates, accurate elucidation of their structures is a prerequisite.

Nuclear magnetic resonance (NMR) spectroscopy has become a reliable tool for structural investigations in such materials. As a spectroscopy technique, NMR is highly sensitive to the electron density about an atom and relies on local structure rather than any long-range order. NMR measurements are typically combined with powder XRD measurements and *ab initio* simulations to obtain refined crystal structures in a technique termed *NMR crystallography*.^12−22^ These refinement procedures, however, often take an expensive iterative approach, as many *ab initio* NMR calculations are repeated until the results converge. Despite advances in computational power and algorithmic efficiencies, the NMR calculation is still expensive and time-consuming.^23^

Machine learning (ML) has increasingly been shown to be useful for providing high-quality predictions for material properties but with orders of magnitude less computational demand.^24−28^ ML techniques have recently been applied to NMR, with most applications focused on organic molecules^29−34^ and a few focused on ^29^Si chemical shift prediction.^35,36^ With ^29^Si NMR databases becoming more widely available^37,38^ machine learning will begin to take a greater role in structural studies. While chemical shifts are useful, as they correlate to the average electronic environment about an atom, shifts are only one piece of the spectrum. The line shape observed in an NMR measurement is described by a tensor, of which the chemical shift is the isotropic part. By ignoring the tensorial nature of the NMR measurement, a myriad of structural information is lost, which is an issue for many previous ML models, as they are only capable of predicting scalar quantities.

Recent advances in the field of geometric deep learning have allowed for prediction of tensorial targets.^39−41^ These models seek to constrain the functions used internally in the model such that only functions that respect the symmetry of the target object can be learned. In the work herein, we present an equivariant GNN model capable of predicting full NMR shielding tensors and demonstrate its capability via ^29^Si nuclear chemical shift tensors in silicate materials.

In the first part of this work we will assess the use of more traditional ML methods to attempt to learn NMR tensor parameters in order to demonstrate that a symmetry-invariant model is insufficient for NMR tensor parameter prediction. We show that all of the symmetry-invariant models trained herein fall short of predicting tensor elements and that instead a symmetry-equivariant model is needed to respect the symmetries of the tensor, as evidenced by a 53% (3.05 ppm vs 6.44 ppm) decrease in mean absolute error compared to invariant models. In the second part of this work we comprehensively assess the performance of our equivariant model. We show that it can learn the full NMR chemical shift tensor to a mean absolute error (MAE) of 1.05 ppm. When converted to the scalar isotropic chemical shift (which previous symmetry-invariant models can predict), our equivariant GNN model outperforms the state-of-the-art model by a large margin, with an MAE of 2.82 ppm versus 5.87 ppm.

We also assess the predicted tensors to show that the equivariant GNN model is capable of learning both tensor magnitude and shape as well as the tensor orientation in a diverse set of silicon local structures.

## Methods

### Dataset

**Figure 1:**
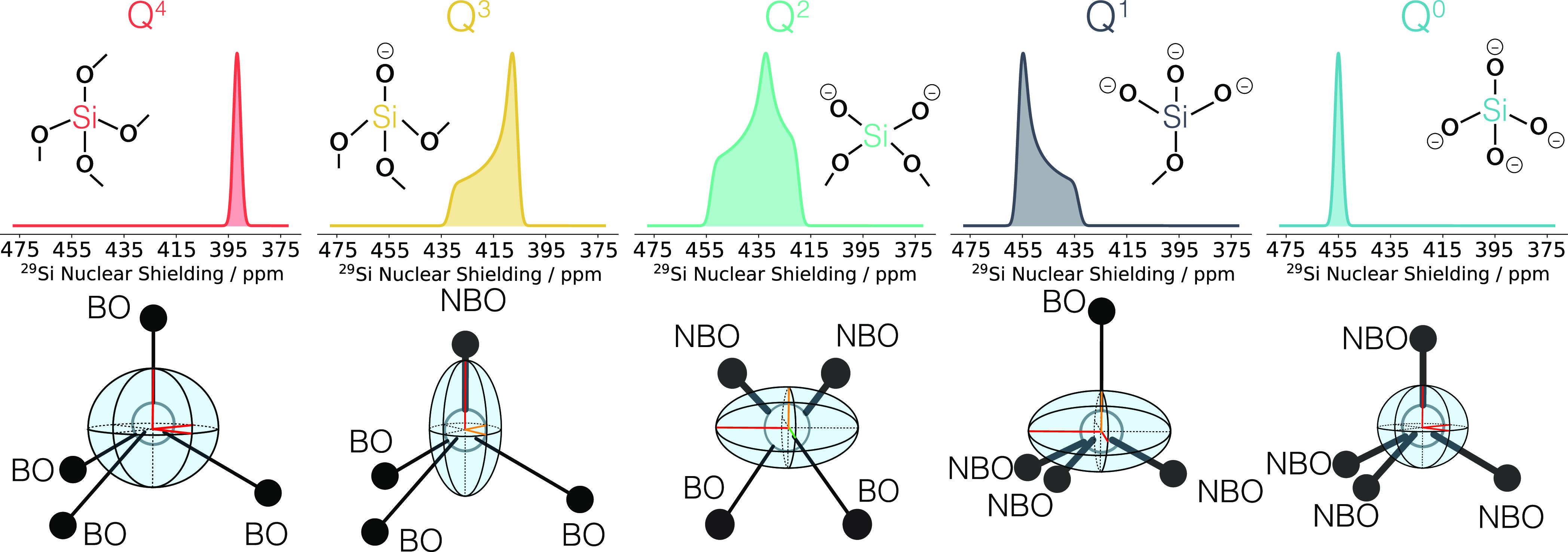
Structural and spectral representations of the NMR chemical shift tensors of different Q^*n*^ species. The structural and geometric variety due to the differences in bridging and nonbridging oxygen bonds results in different chemical environments for each Q^*n*^ and thus different chemical shift tensors.

The ^29^Si NMR dataset used in this study is a subset of *ab initio* NMR chemical shift tensors of relaxed structures calculated by Sun et al.^38^ The dataset is composed of oxygen-coordinated silicon tetrahedral networks consisting of SiO_2_ along with silicates containing group 1 and 2 cations (Li^+^, Na^+^, Mg^2+^, etc.). It contains a wide variety of structures with different numbers of bridging oxygen atoms, *n*, commonly denoted as Q^*n*^, as shown in Figure 1. Each Q^*n*^ species has a different chemical environment and local point-group symmetry due to the differing bond lengths to bridging oxygen (BO) and nonbridging oxygen (NBO), which in effect results in different chemical shift tensor symmetries. In total there are 421 unique silicate structures, consisting of 1387 unique silicon sites. The silicon sites consist of 874 Q^4^ sites, 174 Q^3^ sites, 172 Q^2^ sites, 32 Q^1^ sites, and 97 Q^0^ sites. From each site, the raw calculated rank-2 asymmetric chemical shift tensor was extracted and processed in accordance with each tensor space or tensor convention used in the training of the ML models outlined below.

For each ML model, a train, validation, and test split of 8:1:1 was used. As the dataset consists of multiple types of sites (i.e., each Q^*n*^ species), an attempt was made to stratify the data such that there would be an approximately equal weighting of each Q^*n*^ species in each of the training, validation, and test sets. To remove any opportunity for data leakage, stratification was done at the structure level rather than the site level. Due to structures containing multiple sites, often with different Q^*n*^, each structure was given a label based on whichever *n* was least common in the dataset. For example, in a structure with both a Q^1^ and Q^2^ site, the structure would be labeled as Q^1^ because the dataset consists of a smaller number of Q^1^ sites (32) than Q^2^ sites (172). Structures were then randomly stratified to give roughly equal proportions of each *n* type in each set.

### Chemical Shift Tensor Conventions

Often in the context of NMR, the quantity of interest is a scalar value, the *isotropic chemical shift*, δ^iso^, but it is important to keep in mind that the chemical shift is a tensor quantity, formally an antisymmetric second-rank tensor. Typically, only the symmetric part of the tensor is used, as the symmetric tensor influences the line shapes seen in the NMR spectrum. The distinction between nuclear shielding and chemical shift should also be noted. Nuclear shielding describes the relative change in magnetic field about a nuclear position with respect to the external field and is the quantity calculated during an *ab initio* calculation. In NMR experiments, however, the shielding is not measured directly, and instead, the common practice is to measure the chemical shift as the difference in resonant frequencies between the nucleus of interest and a reference compound.

While nuclear shielding tensors are the typical quantities calculated by *ab initio* methods, we elect to instead use the *absolute chemical shift tensor*, which is the quantity calculated by VASP^42^ and the form originally reported for the database from which the data used herein was obtained.^38^ The formal relationship between the absolute chemical shift tensor, **δ**, and the nuclear shielding tensor, **σ**, is given by^43^
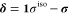
where σ^iso^ is the isotropic nuclear shielding, which is defined as the average of the trace of the nuclear shielding tensor, given as


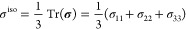
or in the case of the isotropic chemical shift,
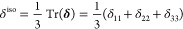
1To convert from the absolute chemical shift tensor to the more familiar referenced chemical shift tensor, **δ**^referenced^, the nuclear shielding tensor of the reference compound, **σ**^ref^, must be calculated and added to the absolute chemical shift tensor:
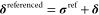
For more information on NMR conventions, the reader is directed to the numerous reviews and textbooks on the topic.^1,44,45^

In symmetry-invariant ML models, an assortment of tensor conventions are used as the training targets. The targets will, in each case, consist of three parameters: the isotropic chemical shift and two additional parameters used to describe the shape of the tensor. The two additional parameters broadly fall into two categories based on the ordering of the principal components of the chemical shift tensor in the principal axis system. The complete list of the conventions used herein is presented in Table 1.

**Table 1:** Chemical Shift Tensor Conventions, Which Fall into Two Categories Based on the Principal Axis Labeling Scheme: Standard Ordering (δ_11_, δ_22_, δ_33_) and Haeberlen Ordering (δ_*XX*_, δ_*YY*_, δ_*ZZ*_)

Standard Ordering: δ_11_ ≥ δ_22_ ≥ δ_33_		
convention name	parameter 1	parameter 2
Maryland (Ωκ)	Ω = δ_11_ – δ_33_	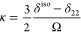 2
axiality/rhombicity (AxRh)	Ax = 2δ_11_ – (δ_33_ + δ_22_)	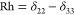 3

Haeberlen Ordering: |δ_*ZZ*_ – δ^iso^| ≥ |δ_*XX*_ – δ^iso^| ≥ |δ_*YY*_ – δ^iso^|		
convention name	parameter 1	parameter 2
Haeberlen (Δδη)	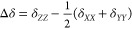	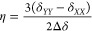 4
Haeberlen (ζη)	ζ = δ_*ZZ*_ – δ^iso^	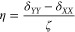 5

Of the conventions used, the Maryland (Ωκ) and Haeberlen (ζη) conventions are the most commonly found, as both are recommended for reporting by IUPAC.^46^ It should be noted that Haeberlen is occasionally presented as (Δδη), but the (ζη) definition is far more common. In addition to Haeberlen (Δδη), another less practiced convention, the axiality/rhombicity (AxRh) convention,^47^ is sometimes used in spin dynamics and spin relaxation theory, as the parameters come from the irreducible spherical tensor expansion of an interaction Hamiltonian. In addition to the four conventions listed in Table 1, we also investigate learning directly on the principal axes (i.e., δ_11_, δ_22_, and δ_33_) in the standard convention to give a total of five conventions investigated for the invariant property predictions.

For our new symmetry-equivariant GNN model, we use both symmetric and asymmetric tensor spaces as targets. In addition to the traditional Cartesian tensors, we also use irreducible representations (irreps) of the Cartesian tensor, **δ**, which are defined as^48^
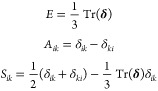
6where *E* is the isotropic part of the tensor, *A*_*ik*_ is the traceless-antisymmetric part of the tensor, *S*_*ik*_ is the traceless-symmetric part of the tensor, and δ_*ik*_ is the Kronecker delta.

Additionally, in the case of the symmetry-equivariant models, a decision must be made on which representation is to be used for interpreting the results. The MAE is satisfactory for a machine to come to an optimized solution but tells us nothing about how well we learned the tensors. A symmetric second-rank tensor has six independent parameters, each of which will need to be assessed. The spherical tensor elements are ideal but are difficult to interpret, as are the tensor indices themselves. The IUPAC-recommended conventions may not be optimal either. The Maryland convention is a descriptor of the line shape as a statistical distribution and is only applicable in specific cases and lacks generalization. The Haeberlen convention is based on the tensor itself and is generally true to the chemical shift tensor; however, the convention requires the ζ parameter to be sign-invariant at η = 1, which creates a degeneracy. Instead, we turn toward a convention recently proposed by Srivastava and Grandinetti^49^ to improve on the issues of the Haeberlen convention. In this convention, ζ and η from the Haeberlen (ζη) convention are first mapped to a polar grid:
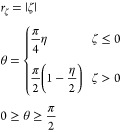
Then the polar grid is mapped onto the first quadrant of the Cartesian grid to give *X* and *Y*, where
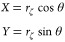
7For interpretation and discussion of the tensors, we use the isotropic chemical shift (magnitude of the tensor), *X* and *Y* (shape/anisotropy of the tensor), and the Euler angles between the laboratory frame and the molecular frame using the *ZYZ* ordering (α, β, γ) (orientation of the tensor).

### Machine Learning Models

#### Graph Neural Networks

**Figure 2:**
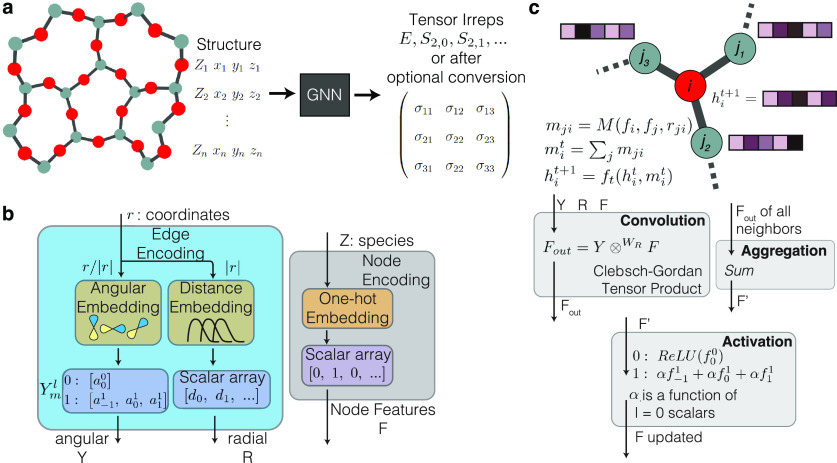
Schematic of an equivariant graph neural network. Each component of the model can have different choices, and the examples here are for the herein-used TFN-based model. (a) Black-box overview of the model, where a structure (represented by a set of atomic numbers, *Z*, and the associated *xyz* coordinates) is passed in as model input and a tensor (which may be represented as tensor irreps or converted to a Cartesian tensor) is obtained as model output. (b) The embedding procedure, in which edge encoding utilizes atomic coordinates to build angular embeddings from a set of spherical harmonics and distance embeddings from a set of learned distance functions. Node encoding utilizes one-hot encoding of the atomic number. (c) The message passing update procedure, in which the edge and node encodings from the neighboring atoms are passed through a convolution filter, aggregated via a sum, and passed through an activation function to update the feature vector on the atom of interest.

In a chemical graph neural network (GNN), shown in Figure 2a, a crystalline structure can be represented as a graph in which each atom is represented by a node, *v*, and relationships between nodes are represented by edges, *e*, which are commonly considered to be chemical bonds, Coulombic interactions, etc. Often the notion of a chemical bond is ill-defined in a crystalline structure, so edges are frequently constructed as all pairwise node connections within some cutoff radius, *r*_cut_, about each atom, taking into account the periodic boundary conditions of the system. All together, the set of nodes, *V* = {*v*_1_, *v*_2_, ..., *v*_*N*_}, and the set of edges, *E* = {*e*_1_, *e*_2_, ..., *e*_*M*_}, make up a graph, *G*(*V*, *E*). To make the graph *G* amenable to machine learning, each node is assigned a feature vector (information of the atomic number and the Cartesian coordinates of the atom in this work). The node data may also be processed to create edge features, *e*_*ij*_, which encode positional information between the two nodes *i* and *j*, as shown in Figure 2b.

The GNNs used in the present work follow the message passing neural network (MPNN) paradigm, in which node features are updated from neighboring nodes in a message passing phase and then the updated features on a node are mapped to a property of interest in a readout phase,^50−53^ as shown in Figure 2c. The objective of the message passing phase is to learn an embedding for each node, *h*, such that unique structural fingerprints for the node are encoded. The message passing typically occurs over a certain number of iterations. During iteration *t*, pairwise interactions between atom *i* and neighboring atoms *j* are summed and processed to produce a message
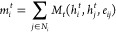
8where *N*_*i*_ is the neighborhood of all atoms surrounding atom *i* within a distance cutoff *r*_cut_, *M*_*t*_ is a learnable function that takes as input the embeddings *h*_*i*_^*t*^ and *h*_*j*_^*t*^ of atoms *i* and *j* as well as their edge data *e*_*ij*_. The embedding of atom *i* is then updated using the message from eq 8:
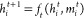
9where *f*_*t*_ is a learnable function. Once the embeddings have been satisfactorily learned, the embedding *h*_*i*_ of atom *i* in the last iteration is then passed to a readout function to produce the property of interest for this atom (NMR tensor of Si atoms in this work).

#### Invariant Graph Neural Networks

We explored rotation-invariant GNNs as example ML models designed for scalar properties. Specifically, the DGL (deep graph library) implementation^54^ of the DimeNet++^55,56^ invariant GNN is selected (we note that DimeNet++ is a strong baseline model in terms of accuracy). The model is customized to allow for the prediction of node properties. A hyperparameter grid sweep was performed to optimize the DimeNet++ model to predict the shift tensor eigenvalues. A total of six separate models are created, each trained on a different shift tensor convention (eqs 1–5) and the standard convention eigenvalues, using the three parameters of the convention as the target. See the Supporting Information for further implementation details and the optimal hyperparameters.

The DimeNet++ model itself is limited to a rotation-invariant mapping from the input structure to the target chemical shift tensor parameters. While the basis functions used in eqs 8 and 9 themselves are rotation-equivariant, the message passing framework is rotation-invariant. Specifically, the coordinate information on each node is only used to initialize distances and angles, which are invariant geometric properties. As a result, DimeNet++ and similar frameworks are limited to predictions of scalar targets, in our case multiple uncorrelated scalar values. The target shift tensor parameters, henceforth called *scalar NMR parameters*, fall under the assumption that the tensor parameters are independent of crystal orientation, as the powder pattern may be used to obtain the scalar NMR parameters. Therefore, the parameters are invariant to rotations.

#### Equivariant Graph Neural Networks

A rotation-equivariant model was created using the Tensor Field Network (TFN)^41^ and e3nn^57^ frameworks, as implemented in the MatTEN^58^ package. In addition to the scalar NMR shift tensor parameters discussed above, this model can directly predict the full chemical shift tensors. An initial equivariant GNN model was implemented (details are given in the Supporting Information) to determine the optimal target on which to train (symmetric vs asymmetric and spherical vs Cartesian tensors). A symmetric spherical tensor target was found to yield the best loss, and a hyperparameter grid search was performed to optimize the equivariant GNN model for the symmetric spherical tensor. To yield a useful model, the symmetric spherical tensor is then converted to a Cartesian tensor, which may be processed as a shift tensor.

An additional rotation-invariant GNN model was similarly created, but this model was trained on the shift tensor eigenvalues. Internally, this model still does message passing using equivariant embeddings (as explained in the following paragraph), but in this case the target is set to the (scalar) eigenvalues instead of the full chemical shift tensor. A hyperparameter grid search was performed to optimize the invariant GNN model (implementation details may be found in the Supporting Information).

In the TFN framework, similarly to DimeNet++, the embedding functions in eq 9 are rotation-equivariant. The TFN framework, however, differs in that the message update function contains convolution filters constrained to the form
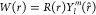
10where *R*(*r*) is a learnable function of the distance between the two nodes and *Y*_*l*_^*m*^(*r̂*) are spherical harmonics taking in the orientation between the nodes. The **W** matrix has the form of a block-diagonal matrix where the blocks correspond to the irreps selected for the network. Additionally, the embedding vectors used in the TFN framework contain blocks corresponding to the irreps. The **W** matrix along with the embedding vectors may then be convolved according to Clebsch–Gordan tensor products to ensure that the symmetry of each irrep is preserved. Thus, the message passing phase of the TFN model uses equivariant embeddings.

### Benchmarking

To the best of our knowledge, no previous model has been proposed to predict full shift tensors. Thus, benchmarking will take place in two steps. The current state-of-the-art model for ^29^Si scalar NMR parameter prediction was introduced by Chaker et al.,^35^ who used linear ridge regression (LRR) over the smooth overlap of atomic positions (SOAP)^59^ features to predict the ^29^Si isotropic chemical shift. We reimplemented this approach using the SOAP features generated by DScribe^60^ and the LRR in scikit-learn.^61^ During the invariant-target benchmark, an LRR-SOAP model will be trained to predict the three eigenvalues of the chemical shift tensor, and all models will be compared on their predictions of the eigenvalues. An LRR-SOAP model will also be trained to predict chemical shift and compared to the models as a benchmark of NMR property prediction. It should be noted that the SOAP formalism has been adapted to be symmetry-equivariant, which may allow SOAP-based models to better predict tensor components.^62^ However, we opted to look only at the invariant SOAP kernel in our benchmarking, as that was the original kernel used by Chaker et al.

Additionally, wherever possible, historic models will be added in the benchmark. These models are neglected in the ML literature for NMR modeling; however, they are widely used in the NMR community, and therefore, benchmarking on such models can provide valuable information. One of these models is the Si–O–Si angle-based ρ model by Engelhardt and Radeglia:^63^
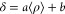
11where *a* and *b* are fitting parameters and ρ is a function of the Si–O–Si bond angle (Ω_Si–O–Si_) that approximates the oxygen s character:
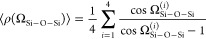
An ^29^Si ζ model introduced by Grimmer et al.^64,65^ and later improved by Jardón Álvarez et al.^66^ is also considered. This model correlates the anisotropy to the difference between the nonbridging-oxygen Si–O bond length and the average bridging-oxygen Si–O bond length in Q^3^ species:
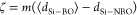
12

## Results and Discussion

### Symmetry-Invariant Learning

The first model types investigated are those trained on rotation-invariant scalar targets. The models are categorized according to the symmetries of the internal embeddings and the final target, labeled as “embedding symmetry” and “target symmetry”, respectively, in Table 2. Of the invariant target models, DimeNet++ performs the best, and LRR-SOAP and the equivariant GNN are on par with each other. However, if a fully equivariant GNN model (equivarant embedding and equivariant target) is used to predict the full shift tensor and then diagonalized to yield the eignevalues, significant improvement over the invariant target models can be achieved. For example, the total MAE is reduced to 3.05 ppm, which is less than half of that from the DimeNet++ model (6.44 ppm). Comparing the equivariant GNN models trained using invariant versus equivariant target symmetry, it is clear that the boost in performance is due to the additional constraint afforded by learning a second-rank tensor rather than three independent scalars. The fully equivariant model will be further discussed in the next section, and here we focus on the invariant models.

**Table 2:** Mean Absolute Error (MAE) for Individual Eigenvalues and Their Averaged Total Prediction Error for Invariant-Target Models^a^

			MAE/ppm			
model	embedding symmetry	target symmetry	total	δ_11_	δ_22_	δ_33_
LRR	invariant	invariant	7.66	8.89	5.86	8.24
DimeNet++	invariant	invariant	6.44	6.45	7.18	5.70
GNN	equivariant	invariant	7.82	9.50	5.23	8.73
GNN	equivariant	equivariant	3.05	3.08	2.84	3.22

a Models are categorized by the symmetry of the embedding functions used in training (training symmetry) and the symmetry of the target at the time the loss was calculated (target symmetry). It should be noted that the last row was not obtained by directly fitting the eigenvalues but rather by fitting the full tensor and then computing the eigenvalues.

The eigenvalues of the shift tensor are not the only invariant targets to consider; one can train ML models to directly predict the NMR parameters in different conventions outlined in Table 1. For this purpose, we selected the DimeNet++ model based on its superior performance in eigenvalue prediction. Five DimeNet++ models were trained (one for each convention), and for ease of comparison, their predictions were converted to the eigenvalues in the standard convention and the two IUPAC-recommended conventions (i.e., the Haeberlen (ζη) convention and the Maryland (Ωκ) convention) using the equations in Table 1. The results are listed in Table 3. We observe that there is no single optimal model, only a “best-in-class” per NMR parameter. For example, the Axiality/Rhombicity convention has the best overall performance but still underperforms on isotropic shift and Maryland Ω values. Furthermore, tensor conventions should be interconvertible. While this is typically true for experimental spectra, we find that it is not the case for ML models trained on individual NMR parameters. For example, the model trained in the Maryland convention performs well when predicting Maryland convention values, but when converted to Haeberlen (ζη), the model ranks the lowest.

**Table 3:** Performance of DimeNet++ Trained on Different Tensor Conventions^a^

training convention	eigenvalues (δ_11_, δ_22_, δ_33_)/ppm	Haeberlen (ζη) convention (ζ/ppm, η)	Maryland (Ωκ) convention (Ω/ppm, κ)	isotropic shift/ppm
Haeberlen (ζη)	(7.53, 6.24, 7.54)	(10.88, 0.24)	(11.56, 0.36)	4.43
Haeberlen Δδ	(7.83, 5.36, 7.85)	(10.91, 0.21)	(12.21, 0.38)	4.07
Maryland (Ωκ)	(6.58, 5.83, 6.10)	(12.51, 0.30)	(7.92, 0.38)	4.12
Axiality/Rhombicity	(5.58, 4.62, 6.39)	(9.71, 0.12)	(7.96, 0.17)	4.21
principal axis system	(6.45, 7.18, 5.70)	(11.02, 0.26)	(7.18, 0.32)	4.23

a For each model, the convention used to train is specified, along with the evaulation errors upon conversion to the Haeberlen (ζη) and Maryland conventions as well as to the eigenvalues and isotropoic shifts. Mean absolute error (MAE) values are reported.

Indeed, it is worthwhile to note that the three NMR parameters predicted are not independent scalars, and when fitted as such, information and internal symmetry constraints are lost. It is therefore not surprising that the use of rotation-invariant models (e.g., DimeNet++) provide inferior results compared to a fully equivariant model. As noted earlier, some conventions are ill-defined and discontinuous for certain values. Additionally, all tensor conventions based on the Cartesian tensor have an issue of explicitly defined axes that can cause confusion when parameters are predicted outside their range, which results in a change of the order of the eigenvalues. A full discussion of the numerical issues that arise when fitting with the tensor conventions can be found in the Supporting Information.

### Symmetry-Equivariant Learning

We now turn our attention toward the rotation-equivariant GNN model, which was shown to significantly outperform the invariant models (see Table 2). Similar to the case of invariant models, there are a variety of output targets available for the equivariant model, among which there is not an *a priori* optimal choice. We focus on using an asymmetric Cartesian tensor, symmetric Cartesian tensor, asymmetric irreps (*E*, *A*_*ik*_, *S*_*ik*_), or symmetric irreps (*E*, *S*_*ik*_) as the target (refer to eq 6). Additionally, the question of which loss function is suitable for learning chemical shift tensors has not, to our knowledge, been investigated for training of ML models. However, there has been substantial work by the MRI diffusion tensor community on optimal tensor metrics for diffusion tensors.^67−72^ The *l*_*n*_ norms offer a good balance of optimizing the shape, magnitude, and orientation of a tensor, and in our case we adopted the *l*_1_ norm as the loss function. We found there is a small benefit to learning on a symmetric tensor versus an asymmetric tensor before symmetrizing. Additionally, there is a minor decrease in both epoch time and the loss when training on an irreps tensor versus a Cartesian tensor. Thus, the optimal space was chosen to be a symmetric irreps tensor using an *l*_1_ norm loss function, and all subsequent results are obtained from models trained using this optimal space.

The best-performing equivariant GNN model exhibits an MAE of 1.05 ppm over the entire chemical shift Cartesian tensor. However, we admit that an MAE calculated for all of the tensor components is challenging to interpret because an asymmetric second-rank tensor has six independent parameters that must be assessed in order to evaluate the performance without loss of information. Therefore, we compare the predicted and DFT-calculated isotropic chemical shift, *X*, *Y*, and Euler angles (α, β, γ) (see Chemical Shift Tensor Conventions for their definition). These parameters were chosen because they provide an intuitive view of the magnitude, shape, and orientation of the tensor, as described above. Additionally, because the shift tensor is very closely linked to the structural point group,^73^ the results are grouped by Q^*n*^ into three clusters reflecting the broad symmetry point group: *T*_*d*_, *C*_3_, and *C*_2_. The results are summarized in Table 4.

**Table 4:** Performance (Reported as Mean Absolute Error) of the Equivariant GNN Model for Each of the Relevant Six Tensor Parameters δ^iso^, *X*, *Y*, α, β, and γ, Organized by Q^*n*^

Q^*n*^	δ^iso^/ppm	*X*/ppm	*Y*/ppm	α/deg	β/deg	γ/deg
Q^4^	1.52	0.52	0.60	60.6	12.2	42.4
Q^3^	3.28	0.80	0.66	47.9	17.9	59.2
Q^2^	6.95	1.71	1.80	39.2	9.0	27.4
Q^1^	8.58	0.85	1.00	1.2	1.3	0.7
Q^0^	1.46	1.27	1.10	49.4	39.9	107
total	2.82	0.85	0.85	53.6	13.9	45.1

**Figure 3:**
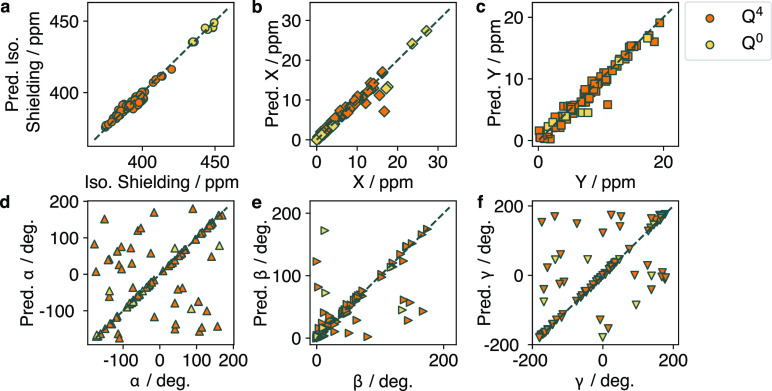
Predicted vs true values of (a) δ^iso^, (b) *X*, (c) *Y*, (d) α, (e) β, and (f) γ for *T*_*d*_-symmetric Q^4^ and Q^0^ sites.

The tetrahedral (*T*_*d*_) sites exhibit low error in the prediction of the magnitude and shape of the tensor, and the tensors are well-predicted by the equivariant GNN model, as shown in Figure 3. While the errors for the Euler angles appear severe in the *T*_*d*_ case, the context of this prediction should be kept in mind. The tensor for a *T*_*d*_ site is highly spherical, showing little to no anisotropy. The axes of a sphere are not unique in these cases, and one would expect a random distribution of the Euler angles. Close inspection of the outlier Euler angles reveals that the high error predictions correspond to sites which are very nearly spherical (small *X* and *Y*). Even in these cases where the orientation of the tensor is less meaningful, the model still provides an accurate prediction of the magnitude and shape of the tensor.

**Figure 4:**
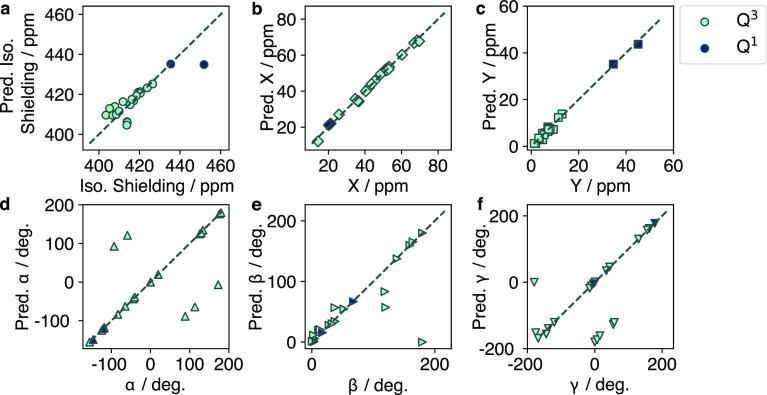
Predicted vs true values of (a) δ^iso^, (b) *X*, (c) *Y*, (d) α, (e) β, and (f) γ for *C*_3_-symmetric Q^3^ and Q^1^ sites.

Moving to the lower-symmetry point group, the *C*_3_-symmetric Q^1^ and Q^3^ sites are responsible for a cylindrically symmetric tensor. The equivariant GNN model shows low error in the anisotropy prediction, as expected due to the strong correlation to the *C*_3_ axis in these sites, as shown in Figure 4. The isotropic shift, however, performs considerably worse than in the tetrahedral case, as is clearly seen in Table 4 (3.28 and 8.58 ppm in Q^3^ and Q^1^, respectively, vs 1.52 and 1.46 ppm in Q^4^ and Q^0^, respectively). Barring a cluster of three data points, the Q^3^ isotropic shift tends to be overpredicted by the equivariant GNN. The sites with overpredicted isotropic shift also correspond to the sites with poorly predicted Euler angles in Figure 4d–f. We speculate that the relatively poor performance of the model is due to the lack of Q^3^ sites in the training data combined with the significant increase in structural diversity compared to the Q^4^ and Q^0^ sites. Furthermore, the structures with anomalous Q^3^ sites trend as outliers in the training set as well and are not well-sampled, which results in poor learning of their structural correlations. In some cases the formula of the material was not seen in the training set, nor were any similar formulas seen, which resulted in a poor extrapolation by the equivariant GNN model. In other cases, the same formula was seen but with minor structural variation, which led to an unfortunate case of the equivariant GNN memorizing the solution poorly.

The situation is made even more extreme in the Q^1^ case due to the low number of samples. The Q^1^ case is, however, fortunate in that despite the poor isotropic shift prediction, the remaining five parameters all show good correlation.

**Figure 5:**
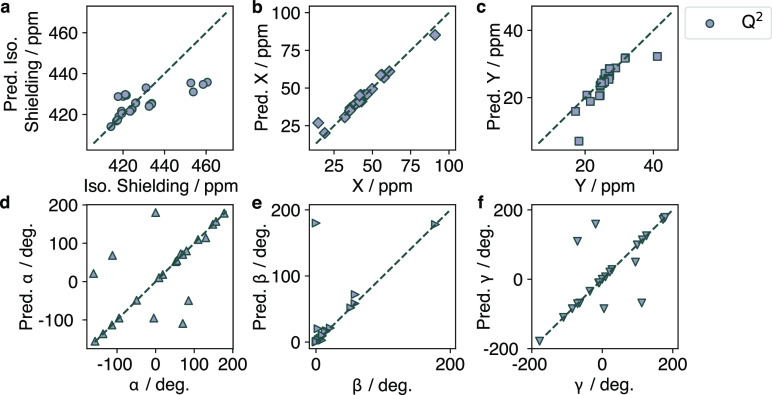
Predicted vs true values of (a) δ^iso^, (b) *X*, (c) *Y*, (d) α, (e) β, and (f) γ for *C*_2_-symmetric Q^2^ sites.

The *C*_2_-symmetric sites exhibit the lowest-order symmetry group and represent a tensor with the shape of an asymmetric spheroid. These sites show the highest error for the isotropic shift, as shown in Figure 5, compared to the *T*_*d*_ and *C*_3_ sites. This is likely a combination of fewer samples in the training data and also the high structural distortion, impairing learning of the isotropic shift (a parameter representing an average). However, the *X* and *Y* values still show that the equivariant GNN model is well-suited for predicting the shape of the tensor, as even in the *C*_2_ case the tensor has rotational axes to which it can correlate. Similar to the *C*_3_ case, the outliers observed in the *C*_2_ case are likely due to the high degree of local structural variation possible. The outliers seen are all poorly sampled in the training set, resulting in the model learning these cases poorly, as was seen with the *C*_3_ sites.

Overall, the equivariant GNN model is able to learn to predict the tensors of silicates, with the best performance being on sites with high symmetry, as summarized in Table 4. Even in cases where the model struggles with isotropic shift or tensor orientation, the shape of the tensor is well-predicted.

It is also instructive to benchmark our equivariant GNN model to historic models and previous state-of-the-art models to ensure that our model constitutes an advance in the field, especially for the domains where previous models were successful. For ^29^Si NMR, the current state-of-the-art model is the LRR-SOAP model proposed by Chaker et al.^35^ to predict isotropic shift.

We trained this model using our dataset to predict isotropic nuclear shifts and obtained an error of 5.87 ppm over the entire dataset (compared to 2.82 ppm with the equivariant GNN over our entire dataset), a Q^4^ MAE of 4.78 ppm (compared to 1.52 ppm with the equivariant GNN), and a Q^3^ MAE of 7.23 ppm (compared to 3.28 ppm with the equivariant GNN) for isotropic shifts. If we instead train the LRR-SOAP on the Q^4^ and Q^3^ chemical shifts separately rather than the entire set, the Q^4^ trained model has an MAE of 4.88 ppm, and the Q^3^ trained model has an MAE of 6.57 ppm, showing that the equivariant GNN improves significantly over state of the art. Additionally, it should be noted that one drawback of the SOAP descriptor is that its size scales with the number of species in the dataset, *N*, as *N*(*N* – 1). Thus, for our entire dataset the SOAP descriptor encoding has a size of 10 980, and while this is not an issue for LRR, the dimensionality may be an issue for other methods especially as the descriptor size is far greater than the dataset size. It should also be noted that the LRR model can only handle scalar values, whereas the main benefit of the equivariant GNN model is in providing the full shift tensor.

It is also important to consider historical analytical expressions, as we lose the descriptive power of analytical expressions when we select an ML method. Determining the coefficient and intercept from eq 11, we obtain *a* = −186.3 ppm and *b* = 477.6 ppm to yield a linear expression with an MAE of 3.60 ppm over the Q^4^ subset. Performing the same analysis with eq 12, we obtain a coefficient of *m* = −1009 ppm Å^–1^ to yield a model with an MAE of 8.77 ppm over the Q^3^ data subset. Compared to the equivariant GNN’s performance with a Q^4^ isotropic shift MAE of 1.52 ppm and a Q^3^ anisotropy MAE of 0.78 ppm, the benefits of the equivariant GNN outweigh the loss of a simple functional form.

## Conclusions

Machine learning approaches are increasingly employed to predict a variety of physical properties, accelerating and expanding access to material data. However, many of those physical properties adhere to inherent constraints, such as symmetry relationships or limits. In the case of tensorial properties, each eigenvalue may be predicted as an independent scalar, but such treatment effectively ignores the underlying symmetry information on the tensor. In this work, we explore the performance of machine learning models that rely on symmetry-invariant fitting procedures and contrast the results with a symmetry-equivariant approach. We find that the NMR tensor parameters cannot be easily learned via symmetry-invariant processes and often contain algebraic structure that makes the learning process more difficult, independent of the tensor convention. By imposing symmetry equivariance, our equivariant GNN model is able to outperform by 53% the symmetry invariant models, demonstrating that handling the tensorial nature of the target is the key to accurately modeling the system.

Examining the results of the equivariant GNN model, we observe that the model is able to accurately predict the tensor, not just in terms of shape and magnitude, but in most cases in the orientation as well. Closer inspection of the cases where the orientation seems to fail shows that these are often the cases of highly spherically symmetric tensors where an orientation is not meaningful. Most surprisingly, the model is able to capture the shape (anisotropy and asymmetry) of the tensor, even in cases where the tensor exhibits very little anisotropy, for example for Q^4^ and Q^0^ sites. Despite the successes of the model, there are still cases where it fails; however, these failures are likely associated with a lack of data in the training set. Future work will be focused toward expanding the dataset, particularly for the Q^*n*^ species, which are less well sampled.

Notably, through the demonstrated work on silicates, it is feasible to predict the full NMR tensor with reasonable accuracy in seconds rather than hours to days as required for *ab initio* calculations. This opens a realm of possibilities from high-throughput screening of materials via comparison to experimental NMR spectra to expediting NMR crystallography refinement procedures.

## Data Availability

Structures and NMR tensors used in the study are hosted on https://contribs.materialsproject.org/projects/lsdi_nmr. The MatTEN code repository is hosted at https://github.com/mjwen/MatTEN.

## Supporting Information Available

Theoretical, implementation, and hyperparameter sweep details for DimeNet++ and equivariant GNN, hyperparameter sweep details for LRR-SOAP, and a discussion on tensor conventions in NMR (PDF)
Jupyter notebook and checkpoint file for a pretrained equivariant GNN model to demonstrate the usage of the model (ZIP)
