# Supplementary material for: Machine Learning Full NMR Chemical Shift Tensors of Silicon Oxides with Equivariant Graph Neural Networks
Source: J Phys Chem A. 2023 Mar 2;127(10):2388–98. doi: 10.1021/acs.jpca.2c07530 (PMC10026072; doi:10.1021/acs.jpca.2c07530)
Supplement: Supplementary file 1 — jp2c07530_si_001.pdf [file jp2c07530_si_001.pdf]

Supporting Information: Machine Learning Full NMR Chemical Shift Tensors of Silicon Oxides with Equivariant Graph Neural Networks

Maxwell C. Venetos,† Mingjian Wen,‡ and Kristin A. Persson*,†,¶

†*Department of Materials Science and Engineering, University of California, Berkeley, CA 94720, United States.*

‡*Department of Chemical and Biomolecular Engineering, University of Houston, Houston, TX 77204, United States.*

¶*Molecular Foundry, Lawrence Berkeley National Laboratory, Berkeley, CA 94720, United States*

*E-mail: kristinpersson@berkeley.edu

DimeNet++ Implementation

Theory

The implementation for the DimeNet++ model follows the implementation of the original model proposed by Klicpera, Groß, and Günnemann[S1] with slight modification to batching to allow for nodal prediction.

The main insight from the DimeNet model is the use of angular representations between atom pair kj and ji, as well as interatomic distance between atoms k and j. Message embeddings in the DimeNet architecture take inspiration from the *particle in a spherical well* problem, in which the wave function used to model the system takes the form

$$\Psi(d, \alpha, \phi) = \sum_{l=0}^{\inf} \sum_{m}^{l} = m = -l(a_{lm} j_l(kd) + b_{lm} y_l(kd)) Y_m^l(\alpha, \phi), \tag{1}$$

where j_l and y_l are spherical Bessel functions of the first and second kind respectively, and Y_m^l are the spherical harmonics. To constrain Eq. (1) to regular solutions b_{lm} is set to 0, and furthermore, to construct a 2-dimensional basis, m is set to 0. Boundary conditions under such constraints are obtained by setting $k = \frac{z_{ln}}{r_{cut}}$ where z_{ln} is the n-th root of the l-order Bessel function. Solution yields the 2-Dimensional Fourier-Bessel basis,

$$\tilde{a}_{SBF,ln}(d, \alpha) = \sqrt{\frac{2}{r_{cut}^3 j_{l+1}^2(z_{ln})}}\, j_l\left(\frac{z_{ln}}{r_{cut}} d\right) Y_m^0(\alpha). \tag{2}$$

A vector representation, $\tilde{\boldsymbol{a}}_{SBF}^{(kj,ji)}$, is then created by taking each $\tilde{a}_{SBF,ln}(d, \alpha)$ with $l \in [0, ..., N_{SHBF} - 1]$ and $n \in [1, ..., N_{SRBF}]$.

To create the radial basis for d_{ji}, degrees of angular dependence are set to zero, i.e. $l = m = 0$, such that the new wave function is $\Psi_{RBF}(d) = a j_0\left(\frac{z_{0n}}{r_{cut}} d\right)$, which is then normalized on $[0, n]$ and with $j_0 = \sin(d)/d$. The radial basis, $\tilde{\boldsymbol{d}}_{RBF}^{(kj)}$ is then given as

$$\tilde{e}_{RBF,n}(d) = \sqrt{\frac{2}{r_{cut}}} \frac{\sin(\frac{n\pi}{r_{cut}} d)}{d}. \tag{3}$$

The $\tilde{\boldsymbol{d}}_{RBF}^{(kj)}$ vector is then created via collecting $\tilde{e}_{RBF,n}(d)$ for $n \in [1, ..., N_{RBF}]$.

Both Eq. (2) and Eq. (3) are not twice differentiable due to a cutoff ar r_{cut} thus an envelop function, $u(d)$, is multiplied to each Eq. (2) and Eq. (3), where the envelop function takes the form

$$u(d) = 1 - 28d^6 + 48d^7 - 21d^8. \tag{4}$$

Messages are then generated from the concatenation of the node embeddings of the atom pair and their radial embedding,

$$\boldsymbol{m}_{ji}^{t+1} = \sigma([\boldsymbol{h}_j^t||\boldsymbol{h}_i^t||\boldsymbol{e}_{RBF}^{(ji)}]\boldsymbol{W} + \boldsymbol{b}) \tag{5}$$

where $||$ denotes the concatenation, σ denotes the sigmoid activation function, and weight matrix $\boldsymbol{W}$ and bias vector $\boldsymbol{b}$ are learnable. From the messages, the node embedding is obtained from the sum of all messages between atom pairs within the cutoff radius, $\boldsymbol{h_i} = \sum_{j \in N_i} \boldsymbol{m_{kj}}$.

In the original implementation, at the time of property read-out the node embeddings are passed through multiple dense layers to generate an atom-wise output, $t_i^{(l)}$. The outputs are then summed to give the final prediction $t = \sum_i \sum_l t_i^{(l)}$, however, in our implementation we refrain from summing the atom-wise predictions and instead give as output the prediction per atom, $t_i = \sum_l t_i^{(l)}$.

Model was trained on the Lawrence Berkeley National Laboratory Lawrencium facility Dell Poweredge 1950 computer running a 3.0GHz AMD 7302P processor node with 64 GB of memory and an Nvidia Tesla A40 GPU.

Hyperparameters

Table S1: Optimal hyperparameters for prediction of ^{29}Si nuclear shielding tensor eigenvalues in the DimeNet++ model.

parameter	optimal value	Grid Search
int embedding size	64	[64, 128]
output embedding size	256	[64, 128, 256]
basis embedding size	8	[8, 16]
r_{cut}	5	[2, 3.5, 5]
num blocks	4	[3, 4, 5]
num spherical harmonic degrees	8	[6, 7, 8]
num radial degrees	6	[6, 7, 8]
learning rate	0.01	[0.1, 0.01, 0.001]
batch size	32	[16, 32, 64]

Eigenn Implementation

Theory

The implementation of the Eigenn model model follows the TFN framework laid forward by Thomas and Smidt *et al.*[S2] to impose rotational equivariance on the model. TFNs use spherical harmonics, Y_m^l and a learned radial function, R_c as filters, F_{cm}. The filter used is restricted such that

$$F_{cm}^{(l_f,l_i)}(\vec{r}) = R_c^{(l_r,l_i)}(r)Y_m^{l_f}(\hat{r}), \tag{6}$$

where l_i and l_f correspond to the spherical harmonic order of the input and output filter respectively, $\vec{r}$ is the vector orientation with respect to the atom center, and r is the magnitude of vector $\hat{r}$.

The filter representation is then stored in a dictionary, denoted $V_{(acm)}^l$, where l denotes the dictionary key and acts to store the filter elements by their spherical harmonic order. a is the point index, i.e. the index of the atom of interest; c is the channel index, i.e. indicies for objects that transform similarly; and m is the representation index, i.e. the starting index for each object.

In order to build a network, each layer must combine the layer input with the filter embedding. The convolution layer may be constructed from the individual filters, F, and the layer input, V, which are then combined with the Clebsch-Gordan coefficients, C, to give a layer

$$\mathcal{L} := \sum_{m_i,m_f} C_{(l_f,m_f)(l_i,m_i)}^{(l_o,m_o)} \sum_{b\in r_{cut}} F_{cm_f}^{(l_f,l_i)}(\vec{r}_{ab})V_{bcm_i}^{l_i}, \tag{7}$$

where, $\vec{r}_{ab}$ is the vector between atoms a and b, and i, f, o indicate input, filter, and output respectively.

To implement the equivariant GNN, we created a network with `4` message passing layers. The radial network was composed of `2` invariant layers consisting of `64` invariant neurons. The convolutional layer was created using an irrep shape of `32x0o + 32x0e + 16x1o +`

16x1e + 8x2o + 8x2e and a nonlinear gate function. The output from the hidden layer was constrained to yield irreps 16x0e + 8x1e + 4x2e.

To create the embeddings, a species embedding vector of size 16 was used along with 12 radial basis functions and edge irreps of the shape 0e + 1o + 2e. The cutoff neighborhood was set to 5.0 Å.

During training, a batch size of 16 is used with the Adam optimizer and a learning rate 0f 0.01 and an l_1-norm loss function. Model was trained on the Lawrence Berkeley National Laboratory Lawrencium facility Dell Poweredge 1950 computer running a 3.0GHz AMD 7302P processor node with 64 GB of memory and an Nvidia Tesla A40 GPU. The final optimized model had 306928 parameters.

Hyper-parameters

Table S2: Optimal hyperparameters for prediction of ^{29}Si nuclear shielding tensor in the equivariant Eigenn model.

parameter	optimal value	Grid Search
Number of Layers	4	[3, 4, 5]
Species Embedding Dimension	16	[16, 32]
Number Radial Basis	12	[8, 12, 16]
r_{cut}	5	[2, 3.5, 5]
Number of Invariant Layers	2	[1, 2]
Number of Invariant Neurons	64	[16, 32, 48, 64]
Batch Size	16	[16, 32, 64]
Learning Rate	0.001	[0.1, 0.01, 0.001]

Table S3: Optimal hyperparameters for prediction of ^{29}Si nuclear shielding tensor in the invariant Eigenn model.

parameter	optimal value	Grid Search
Number of Layers	5	[3, 4, 5]
Species Embedding Dimension	16	[16, 32]
Number Radial Basis	12	[8, 12, 16]
r_{cut}	5	[2, 3.5, 5]
Number of Invariant Layers	2	[1, 2]
Number of Invariant Neurons	16	[16, 32, 48, 64]
Batch Size	64	[16, 32, 64]
Learning Rate	0.01	[0.1, 0.01, 0.001]

LRR-SOAP Hyperparameters

Table S4: Optimal hyperparameters for prediction of ^{29}Si nuclear shielding tensor eigenvalues in the LRR-SOAP model.

parameter	optimal value	Grid Search
SOAP n_{max}	2	[2, 4, 6, 8]
SOAP l_{max}	8	[2, 4, 6, 8]
SOAP r_{cut}	3.5	[2, 3.5, 5]
LRR α	1	[0.01, 0.02, ... , 1.00]

Failures of an Tensor Conventions

Its well known that tensor conventions in NMR pose somewhat of an issue. It is typically recommended to use the Haeberlen or Maryland convention to report a tensor, but these conventions are often defined in an algebraically dangerous manner and may have features which make machine learning difficult. The two Haeberlen conventions have a discontinuity at $\zeta = \Delta\sigma = 0$ splits the space in two and crossing the discontinuity corresponds to an axis flip, which is discussed further below. Additionally, $\eta = 1$ creates a degeneracy making learning near these points difficult. The Ohio XY convention is an attempt to alleviate some of the issues of the Haeberlen convention by compressing the discontinuity to a single point, however, by taking the magnitude of ζ we are no longer able to convert back to ζ, and is no longer suitable for machine learning. The Maryland convention also has a discontinuity at $\Omega = 0$, however, this discontinuity is at the edge of the data rather than the middle of the data, resulting in a somewhat easier to learn space. The AxRh convention (and similarly the eigenvalues themselves) improves on the others in that there is no discontinuity, however, every convention derived from the Cartesian tensor suffers from an axis switching problem. The axis switching bounds the data at $\eta \in [0, 1]$ in the Haeberlen convention, $\kappa \in [-1, 1]$ in the Maryland, and between the x-axis and y=x line in the positive quadrant in the AxRh convention.

These bounds and infinite discontinuities are not differentiable and result in difficult to learn regions for the ML algorithms. This is especially an issue in early phases of the model where errors are large and may yield unphysical predictions as the model has not correctly learned the output space.

There is an additional feature in table 3 of the main text which shows the dangers of the Cartesian tensor conventions and is the result of the axis switching that often arises when one converts from the Haeberlen convention to the Maryland convention. Models that perform well in one convention tend to perform very poorly once switched to the other convention. For example, the model trained on the Maryland convention performs vary well when the

metric considered is the Maryland convention, however, it becomes the worst performing model when converted to the Haeberlen ($\zeta\eta$) or ($\Delta\sigma\eta$) conventions. This result appears to violate the assumption that one may inter-convert between the different conventions. This issue arises when converting between conventions using two different axes orderings.

The cause of this issue is two-fold. Due to the axis switching in the Haeberlen convention as one moves from negative to positive values of ζ, writing out the Haeberlen ($\zeta\eta$) definition in terms of the standard convention yields two different equations, one in which $\zeta > 0$:

$$\begin{gathered} \zeta_{>0} = \sigma_{11} - \sigma^{iso} = \tfrac{1}{3}(2\sigma_{11} - \sigma_{22} - \sigma_{33}) \\ \eta_{\zeta>0} = \tfrac{\sigma_{22}-\sigma_{33}}{\zeta}, \end{gathered} \tag{8}$$

and one in which $\zeta < 0$:

$$\begin{gathered} \zeta_{<0} = \sigma_{33} - \sigma^{iso} = \tfrac{1}{3}(2\sigma_{33} - \sigma_{22} - \sigma_{11}) \\ \eta_{\zeta<0} = \tfrac{\sigma_{22}-\sigma_{11}}{\zeta}. \end{gathered} \tag{9}$$

The second issue is with respect to the errors of the model prediction and the error propagation through the transformation from one axis ordering scheme to another. Using the error propagation equation

$$\delta_Y^2 = \sum_{i=1,2,3} \left(\frac{\partial f}{\partial \sigma_{ii}}\right)^2 \delta_{\sigma_{ii}}^2, \tag{10}$$

where δ_Y is the error in parameter Y which is a function, f, of σ_{11}, σ_{22} and σ_{33}. Illustrating the case for the Haeberlen convention, substituting Eq. (8) and Eq. (9) into Eq. (10) yields

$$\delta_{\zeta>0}^2 = \frac{4}{9}\delta_{\sigma_{11}}^2 + \frac{1}{9}\delta_{\sigma_{22}}^2 + \frac{1}{9}\delta_{\sigma_{33}}^2 \tag{11}$$

and

$$\delta_{\zeta<0}^2 = \frac{1}{9}\delta_{\sigma_{11}}^2 + \frac{1}{9}\delta_{\sigma_{22}}^2 + \frac{4}{9}\delta_{\sigma_{33}}^2. \tag{12}$$

From inspection of Eq. (11) and Eq. (12) one can see that σ_{11} and σ_{33} have different effects

on the ζ error for the different values of ζ. This case arises in ML as we have not put any constraints on the loss function to guide σ_{11} and σ_{33} to have the same error.

While the Haeberlen-based models tend to have poor performance metrics compared to other models, the training in the Haeberlen convention did have one major benefit. The difficult manifold learned for the Haeberlen conventions forced the models to learn a mapping between structure and tensor such that the error on the σ_{11} and σ_{33} eigenvalues were approximately equal to each other. The approximately equal errors for the two components, however, came at the cost of a significantly higher error in the eigenvalues when compared to the conventions that do not have such a difficult topology such as the Maryland and standard convention. While it may be beneficial to have additional constraints on the loss or force the model to learn a better representation through a difficult topology, a more elegant solution may be to impose additional symmetry constraints on the model its-self to predict a full tensor rather than three independent scalars.

We also find that the NMR tensor parameters may be unsuitable as metrics to rank performance for ML models. When assessing the utility of a model for experimental use the MAE of the full tensor may not be a helpful metric to visualize whereas comparing the Haberlen $\zeta\eta$ or Maryland $\Omega\kappa$ parameters may be more attractive options as they are what is ultimately used by spectroscopists.

The most striking feature shown in Fig. S1 is the Q^4 and Q^0 anisotropy which shows a $y = -x$ correlation for some points close to $\zeta = 0$ which have been similarly reported.[S3] Analysis of these points that exhibit this behavior often shows nearly perfect predictions of the tensor and tensor eigenvalues. Close inspection reveals that despite such good predictions of eigenvalues, the relative position of the isotropic shift in relation to the intermediate eigenvalue can shift the sign of ζ. Even if the difference between predicted values is small, fractions of a percent, if the isotropic shielding is larger than σ_{22} when it should be smaller than it or vice-versa, then the resulting error in ζ is substantial. Due to the fragility of the Haeberlen $\zeta\eta$ convention we do not recommend using it as a metric unless care is taken to

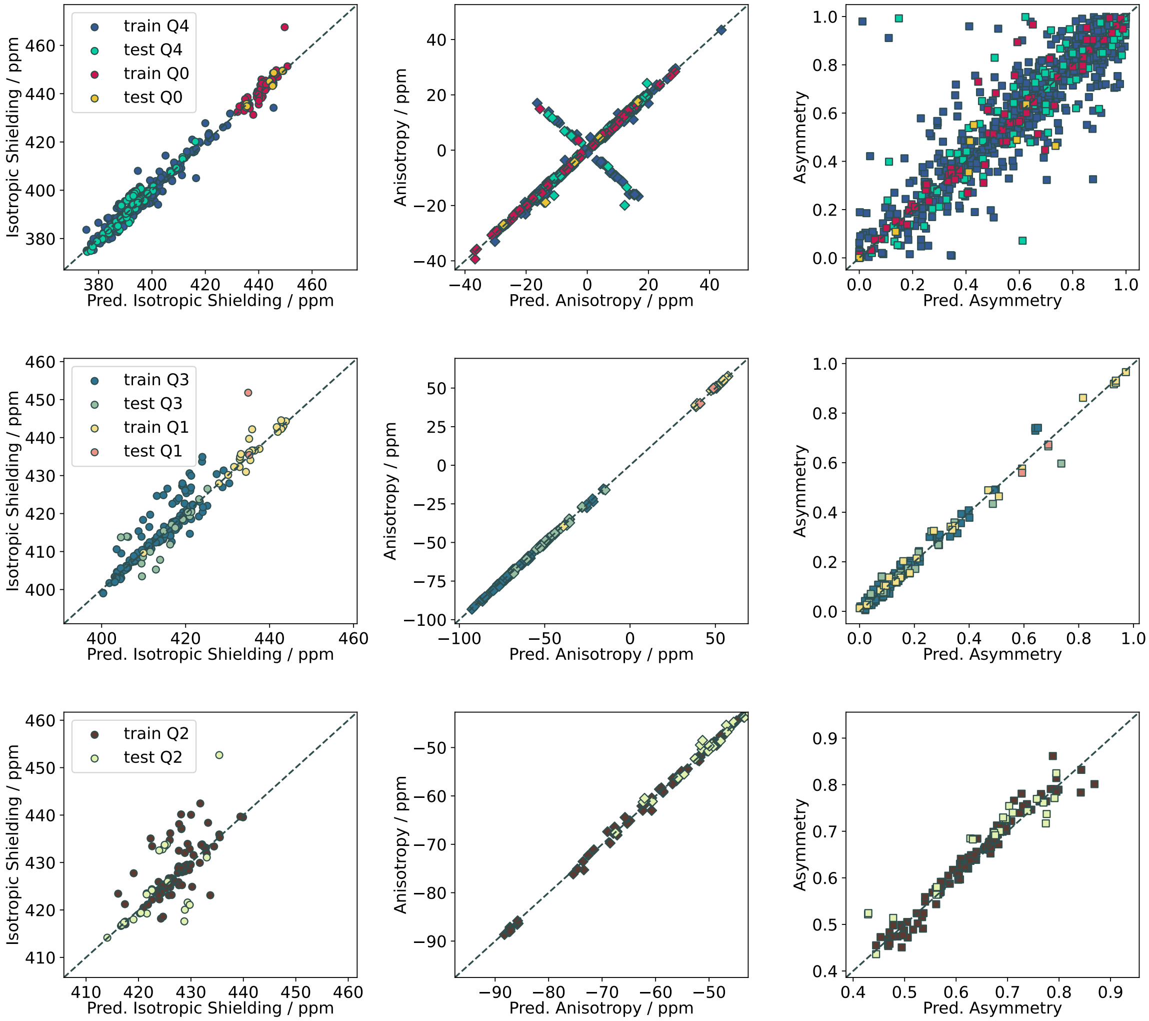

Figure S1: Predicted and true Haeberlen ($\zeta\eta$) values in training and test sets for the different point groups.

avoid this issue.

Similarly, the Q^4 and Q^0 η (and κ) parameters show significant error. This parameter is ill-suited to describe such spherically symmetric tensors and errors may be high for the smallest numerical inaccuracies. Furthermore, η has little influence on spectral fitting for these sites as the parameter describes the skewness of the line shape and for Q^4 and Q^0 sites, which exhibit tight line shapes, the differences in the skewness are difficult to detect. Overall, the isotropic shielding along with XY are more robust at describing the tensor and should be used as metrics rather than the normal tensor conventions.

References

(S1) Klicpera, J.; Groß, J.; Günnemann, S. Directional Message Passing for Molecular Graphs. *CoRR* **2020**, *abs/2003.03123*.

(S2) Thomas, N.; Smidt, T.; Kearnes, S.; Yang, L.; Li, L.; Kohlhoff, K.; Riley, P. Tensor field networks: Rotation- and translation-equivariant neural networks for 3D point clouds. 2018; https://arxiv.org/abs/1802.08219.

(S3) Sun, H.; Dwaraknath, S.; Ling, H.; Qu, X.; Huck, P.; Persson, K.; Hayes, S. Enabling materials informatics for 29Si solid-state NMR of crystalline materials. *npj Computational Materials* **2020**, *6*, 53.
